# Microsatellite instability and mismatch repair deficiency in bladder urothelial carcinoma: a Tunisian single-center study

**DOI:** 10.1186/s43046-025-00279-x

**Published:** 2025-05-26

**Authors:** Ahlem Bdioui, Mariem Akkari, Maroua Krifa, Yosra Souiden, Ethmane Sleimane, Wafa Mokni, Nada Ben Lazrek, Sarra Mestiri, Sihem Hmissa, Nabiha Missaoui

**Affiliations:** 1 Research Laboratory Lr21es03, Oncogenesis and Tumor Progression, Medicine Faculty of Sousse, University of Sousse, Sousse, Tunisia; 2 Pathology Department, Sahloul University Hospital, Sousse, Tunisia; 3 Pathology Department, Farhet Hached University Hospital, Sousse, Tunisia

**Keywords:** Urothelial carcinoma, Bladder, DMMR, MSI, Immunohistochemistry, PCR

## Abstract

**Background:**

Microsatellite instability (MSI) and deficiency in the human mismatch repair (MMR) system are critical drivers of genomic instability in various cancers. Tumors exhibiting MSI and MMR deficiency (dMMR) have prognostic implications and are associated with differential responses to immune checkpoint inhibitors. Given their key roles in tumorigenesis, investigating MMR protein expression and MSI in urothelial cancer of the bladder is essential to improve therapeutic strategies and deepen understanding of its molecular features. This study aimed to assess MMR protein expression and MSI in primary urothelial carcinoma of the bladder and to evaluate their associations with clinicopathological characteristics.

**Methods:**

A total of 49 primary urothelial carcinomas were analyzed for MMR expression using immunohistochemistry, and dMMR tumors underwent further analysis for MSI status using the markers of the Bethesda panel (BAT25, BAT26, D2S123, D5S346, and D17S250). The MMR expression and MSI findings were associated with clinicopathological parameters.

**Results:**

dMMR was identified in two high-grade urothelial carcinomas (4.1%), while the remaining cases demonstrated proficient MMR. Both dMMR tumors showed impaired immunoreactivity, with one tumor displaying a simultaneous loss of the MLH1/PMS2 heterodimer and the other showing isolated MSH6 loss. MSI analysis revealed instability in BAT26 in the MLH1/PMS2-deficient tumor and at D17S250 in the MSH6-deficient tumor. Both tumors exhibited low-level MSI (MSI-L). No relevant associations were found between MMR/MSI status and clinicopathological features (*p* > 0.05).

**Conclusions:**

The identification of MSI-L and MMR deficiency in only two samples underscores the rarity of MSI in urothelial carcinoma among Tunisian patients. These findings emphasize the need for larger, multi-center studies to elucidate the MSI/dMMR molecular and clinical implications in bladder carcinoma.

## Introduction

Bladder cancer is the ninth most frequently diagnosed malignancy globally and ranks as the sixth primary cause of cancer-induced mortality among men [1]. In Tunisia, bladder cancer is the fourth most prevalent cancer, with an incidence of 9.4 per 100,000 individuals [2, 3]. Almost 75% of newly diagnosed bladder cancer cases are classified as non-muscle invasive (pTa) or minimally invasive (pT1). These tumors are generally associated with favorable long-term survival rates but have a high recurrence risk, affecting approximately 60% of patients. Management of non-muscle invasive bladder cancer typically includes transurethral resection followed by 1 to 3 years of intravesical chemotherapy or immunotherapy. In contrast, 25% of bladder cancers are muscle-invasive (stage pT2 or higher) and are linked to a significantly poorer prognosis, with an overall mortality rate approaching 50%. Treatment for muscle-invasive bladder cancer often involves more aggressive therapeutic strategies, such as radical cystectomy with urinary reconstruction or radiotherapy, combined with or without chemotherapy [4–6]. Additionally, systemic therapies, particularly immune checkpoint inhibitors, have demonstrated considerable efficacy in select patient populations [7]. This epidemiological and clinical landscape highlights the need for tailored therapeutic approaches and ongoing research to improve outcomes in both non-muscle invasive and muscle-invasive bladder cancer cases.

The development and progression of bladder cancer are complex, multistep processes influenced by both genetic predispositions and environmental exposures. Central to this process are the roles of oncogenes, tumor suppressor genes, and genes involved in DNA damage repair [8]. A critical component in preserving genomic stability is the mismatch repair (MMR) system, which identifies and repairs replication errors such as base–base mismatches and insertion-deletion loops. The MMR system relies on key proteins, including MLH1 (MutL homolog 1), PMS2 (PMS1 homolog 2), MSH2 (MutS homolog 2), and MSH6 (MutS homolog 6), which operate as heterodimers to maintain genomic integrity. Specifically, the MSH2-MSH6 complex (hMutSα) identifies and binds to mismatched DNA base pairs. This recognition facilitates the recruitment of the MLH1-PMS2 complex (hMutLα), which mediates the removal of the erroneous DNA segment and the subsequent synthesis of a corrected strand [9–11]. Consequently, the loss of MLH1 or MSH2 leads to the degradation of these complexes, compromising the MMR system and facilitating genomic instability [12]. Deficiencies in the MMR system result in microsatellite instability (MSI), a condition characterized by mutation accumulation, particularly within repetitive DNA sequences, and a high frequency of somatic mutations, primarily frameshift mutations [13, 14]. This genetic instability plays a key role in the pathogenesis of diverse cancers, most notably colorectal and endometrial cancers [15]. Furthermore, MSI is frequently found in malignancies linked to Lynch syndrome, also referred to as hereditary non-polyposis colorectal cancer (HNPCC).

To enable accurate MSI assessment, the reference panel endorsed by the International Workshop on MSI and RER phenotypes in cancer detection and familial predisposition, held in Bethesda, defines MSI-high (MSI-H) tumors as those exhibiting instability in two or more markers, while MSI-low (MSI-L) tumors are characterized by instability in just one marker [16]. This standardized panel comprises two mononucleotide repeats (BAT-25 and BAT-26) and three dinucleotide repeats (D2S123, D5S346, and D17S250) [17]. By using this reference panel, clinicians can accurately classify tumors based on their MSI status, which in turn enhances the precision of treatment approaches, particularly in terms of immune checkpoint inhibitors [18].

To further explore the molecular mechanisms underlying bladder oncogenesis, our study investigates MMR protein expression and MSI phenotype in urothelial carcinoma patients, which may offer valuable insights for refining therapeutic approaches and improving clinical outcomes.

## Materials and methods

### Study samples

The study was conducted at the Laboratory of Oncogenesis and Tumor Progression, Faculty of Medicine, Sousse, in close collaboration with the Pathology Department at Sahloul University Hospital, Sousse, Tunisia. Approval for the use of archival material for research purposes was obtained from the local Human Ethics Committee at Sahloul University Hospital, and the study was conducted in accordance with the principles outlined in the Declaration of Helsinki.

Forty-nine bladder tumors diagnosed between 2020 and 2022 were included in this retrospective study. Urothelial carcinoma samples were sourced from the Urological Surgery Department, diagnosed at the Pathology Department of Sahloul University Hospital, Sousse (Tunisia), and registered in the Cancer Registry of Central Tunisia during the study period (2020–2022).

Patient clinicopathological characteristics were extracted from the pathology reports and clinical records archived in the Pathology Department at Sahloul University Hospital, Sousse (Tunisia). These data included age at diagnosis, gender, smoking status, personal and family medical history, circumstances of cancer detection, tumor localization and size, histological type, TNM stage, tumor differentiation, nodal and vascular invasion, and the presence of metastasis. Patient follow-up data and clinical outcomes were obtained from the Cancer Registry of Central Tunisia.

Histological diagnoses for all cases were reviewed by two surgical pathologists from the Pathology Department at Sahloul University Hospital, Sousse (Tunisia), using hematoxylin and eosin-stained sections (AB and SH), according to the 2022 World Health Organization (WHO) classification [19]. Tumor staging was performed in accordance with the eighth edition of the AJCC cancer staging guidelines [20]. One or two tissue blocks from each tumor sample were selected for confirmation of the histopathological diagnosis and for subsequent immunohistochemistry and molecular analysis. All tissues were routinely preserved in 10% buffered formalin and subsequently embedded in paraffin.

### Immunohistochemistry

MMR protein immunostaining was performed immunohistochemistry on 4-µm-thick whole-slide sections of archived tissues, as recently detailed [20]. In short, after antigen retrieval and blocking the endogenous peroxidases, slides underwent incubation with primary mouse monoclonal antibodies (anti-MLH1, anti-PMS2, anti-MSH2, and anti-MSH6) for 30 min (Table 1). The immunoreactivity was recognized by using the Leica BOND-MAX automated system (Leica Biosystems, UK) and the Bond Polymer Refine Detection kit (DS9800, Leica Biosystems, UK) according to the manufacturer’s instructions. The immunostaining was visualized with diaminobenzidine, and the sections were briefly counterstained with Hematoxylin and mounted [21].

**Table 1:** Primer sequences and annealing temperatures for MSI marker amplification [16]

Marker	Primer sequences (5′−3′)	Annealing temperature
BAT25	TCGCCTCCAAGAATGTAAGT TCTGGATTTTAACTATGGCTC	48 °C
BAT26	TGACTACTTTTGACTTCAGCC AACCATTCAACATTTTTAACC	48 °C
D5S346	AAACAGGATGCCTGCCTTTA GGACTTTCCACCTATGGGAC	50 °C
D2S123	ACTCACTCTAGTGATAAATCGGG AGCAGATAAGACAAGTATTACTAG	53 °C
D17S250	GGAAGAATCAAATAGACAAT GCTGGCCATATATATATTTAAACC	48 °C

Two pathologists independently assessed the MMR protein expression by evaluating nuclear staining in both normal surrounding cells (inflammatory and stromal cells) and malignant cells. Tumors were categorized as proficient MMR (pMMR) if all four MMR proteins showed positive nuclear expression in tumor cells, or as deficient MMR (dMMR) if there was a loss of immunoreactivity in at least one MMR protein.

### Microdissection and DNA extraction

An experienced surgical pathologist first identified and marked regions with approximately 80% tumor cell density on a representative hematoxylin and eosin-stained section. Non-malignant tissue was carefully excised using a sterile needle and collected in a sterile tube. A separate sterile needle was then used to collect the tumor tissue, which was placed into another sterile tube.

DNA was isolated from both tumor and normal formalin-fixed, paraffin-embedded (FFPE) tissue samples using the QIAamp DNA FFPE Tissue Kit (Qiagen, Hilden, Germany) as instructed by the manufacturer. The extracted DNA underwent quality assessment via polymerase chain reaction (PCR) targeting a 268-base pair (bp) fragment of the *β*-*globin* gene (S: 5′-CAACTTCATCCACGTTCACC-3′ and F: 5′-GAAGAGCCAAGGACAGGTAC-3′) [22].

### MSI analysis

Cases displaying any MMR loss via immunohistochemistry underwent MSI testing by analyzing paired normal and tumor DNA using the Bethesda National Cancer Institute consensus panel markers BAT25, BAT26, D5S346, D2S123, and D17S250 [16]. Primer sequences are listed in Table 1. A total of 200 ng of template DNA was amplified by PCR in a final reaction volume of 25 µl, containing 1 × PCR buffer (10 mM Tris, pH 8.3, 50 mM KCl), 2.5 mM MgCl_2_, 0.2 mM each deoxynucleotide triphosphate, 0.5 mM each primer, and 1 unit of Taq DNA polymerase (Qiagen, Hilden, Germany). Amplification was carried out using a Bio-Rad T100 thermocycler (Bio-Rad, Marnes-la-Coquette, France) under the following conditions: initial denaturation at 95 °C for 5 min, followed by 40 cycles of 1 min at 95 °C, 1 min at the annealing temperature specified in Table 1, and 1 min at 72 °C, with a final extension at 72 °C for 10 min. PCR products were separated on a 29:1 acrylamide/bisacrylamide gel via electrophoresis, stained with ethidium bromide, and visualized under UV light using the GelDoc2000 System (Bio-Rad, Marnes-la-Coquette, France).

MSI was characterized by the detection of additional bands or band shifts in the tumor DNA compared to the corresponding normal DNA. All analyses were performed in duplicate with independent PCR reactions to minimize errors caused by the preferential amplification of one allele. Tumors were classified as microsatellite stable (MSS) if none of the markers showed instability, MSI-L if a single marker was unstable, and MSI-H if two or more markers showed instability [16].

### Statistical analysis

Statistical analysis was performed using SPSS version 27.0 (SPSS Inc., Chicago, IL, USA). Demographic, clinical, and histopathological characteristics of bladder cancer patients were compared between groups using the chi-square test and Fisher’s exact test. Categorical variables are presented as frequencies (*n*) and percentages (%). A *p*-value of ≤ 0.05 was considered statistically significant.

## Results

The survey involved 49 patients with bladder carcinoma, comprising 45 men (91.8%) and 4 women (8.2%), resulting in a male-to-female ratio of 11:1. The clinicopathological characteristics of the patients are summarized in Table 2. Patients’ ages ranged from 50 to 94 years, with a median age of 70 years. Smoking data were available for 16 patients, all of whom were tobacco users.

All cases were classified as muscle-invasive carcinoma. According to the 2017 TNM classification, 85.7% of the cases were high-grade, while 14.3% were low-grade. Approximately 69.4% of the tumors were classified as pT3 or higher. Lymph node metastases were present in 46.9% of cases, and vascular emboli and perineural invasion were observed in 59.2% and 44.9% of cases, respectively.

**Fig. 1:**
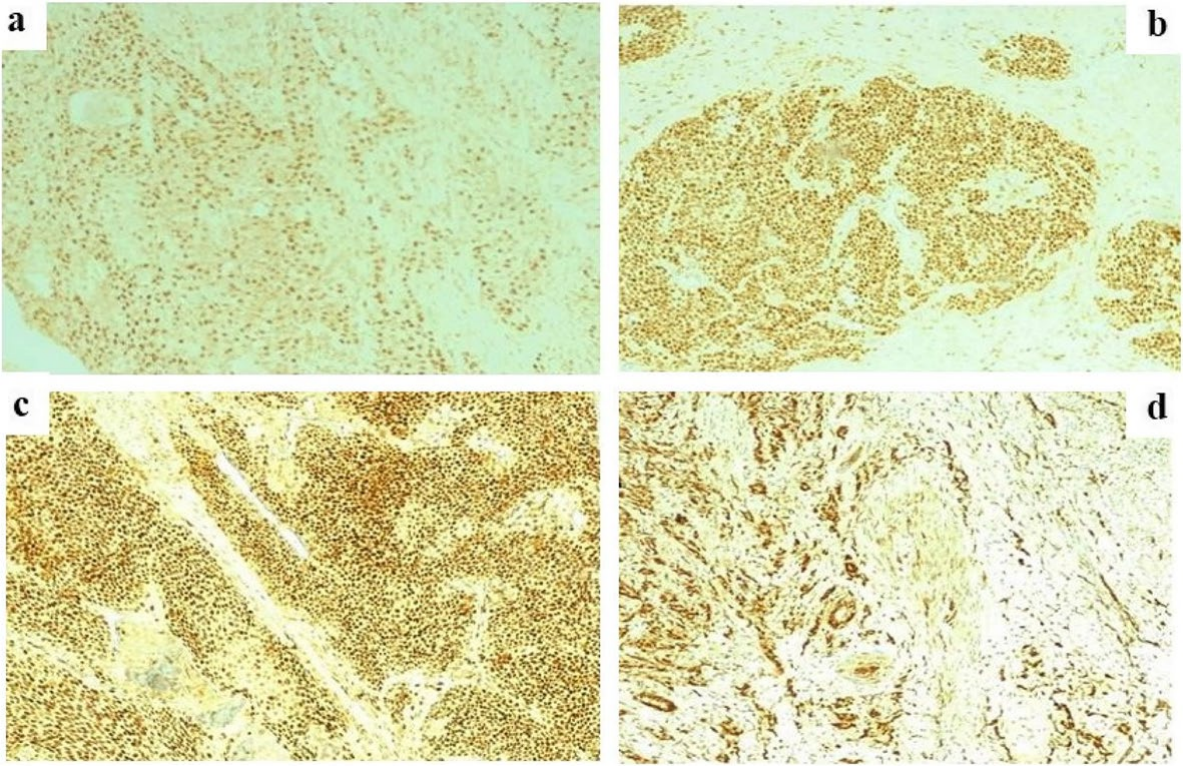
Representative immunohistochemical staining of MMR proteins in bladder urothelial carcinoma. **a** MLH1 expression. **b** MSH2 expression. **c** MSH6 expression. **d** PMS2 expression. (original magnification, × 100)

**Fig. 2:**
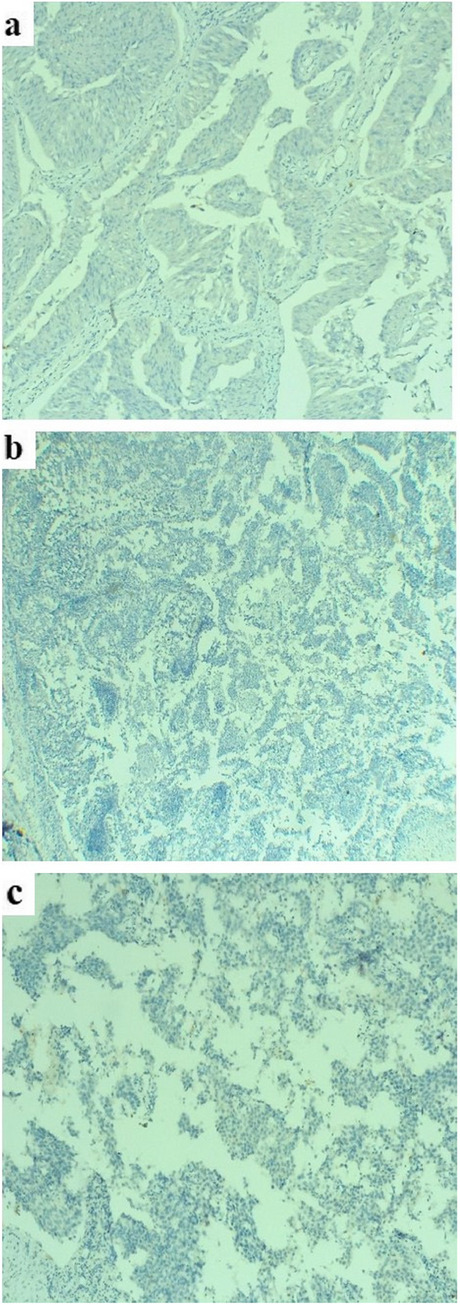
Representative immunohistochemical staining of MMR deficiency in urothelial carcinoma of the bladder. **a** Expression lack of MLH1. **b** Expression lack of PMS2. **c** Expression lack of MSH6 (original magnification, × 100)

Immunohistochemical analysis of MMR expression revealed a dMMR profile in two cases (4.1%) and a pMMR status in the remaining 47 cases (95.9%) (Fig. 1). MMR deficiency was characterized by the concurrent loss of the heterodimeric pair MLH1/PMS2 in one tumor and the exclusive loss of MSH6 in the other (Fig. 2).

Both dMMR patients were male, with a median age of 69 years, and both had high-grade urothelial tumors. Statistical analysis revealed no significant differences in clinicopathological parameters between the dMMR and pMMR groups (*p* > 0.05) (Table 2). Low-grade tumors were observed exclusively in the pMMR group, while high-grade tumors were present in both groups, with slightly higher perineural involvement in the dMMR group.

**Table 2:** Clinicopathological characteristics of patients with bladder carcinoma according to MMR status

Characteristic	Total (*N* = 49)	pMMR (*N* = 47)	dMMR (*N* = 2)	*p*-value
Age (years(range))	70.18 (50—94)	70.43(50—94)	69 (56—82)	0.84
Gender (M/F)	45/4	43/4	2/0	0.67
Smoking	16	16	0	-
Tumor grade				
Low-grade	7 (14.3%)	7	0	0.55
High-grade	42 (85.7%)	40	2	
pT Stage				
Muscle-invasive T2	15 (30.6%)	14 (29.8%)	1 (50.0%)	
Muscle-invasive T3	15 (30.6%)	15 (31.9%)	0 (0.0%)	0.31
Muscle-invasive T4a	19 (38.8%)	18 (38.3%)	1 (50.0%)	0.86
Lymph node metastases pN				
N0	26 (53.1%)	25 (53.2%)	1 (50.0%)	
N1	8 (16.3%)	7 (14.9%)	1 (50.0%)	0.36
N2	10 (20.4%)	10 (21.3%)	0 (0.0%)	0.53
N3	1 (2.0%)	1 (2.1%)	0 (0.0%)	0.84
Nx	4 (8.2%)	4 (8.5%)	0 (0.0%)	0.69
Vascular emboli				
Presence	29 (59.2%)	28 (59.6%)	1 (50.0%)	0.78
Absence	20 (40.8%)	19 (40.4%)	1 (50.0%)	
Perineural sheaths				
Presence	22 (44.9%)	21 (44.7%)	1 (50.0%)	0.88
Absence	27 (55.1%)	26 (55.3%)	1 (50.0%)	

*pMMR* proficient mismatch repair, *dMMR* deficient mismatch repair, *p* < 0.05: statistical significance

The two samples with a dMMR profile underwent MSI testing, which revealed distinct patterns. The first patient, a 56-year-old diagnosed with high-grade urothelial carcinoma of the bladder classified as pT4aN1, exhibited concurrent loss of MLH1 and PMS2. By contrast, the second patient, an 82-year-old with high-grade bladder cancer staged as pT2N0, demonstrated isolated loss of MSH6. MSI analysis revealed instability at BAT26 in the MLH1/PMS2-deficient tumor and at D17S250 in the MSH6-deficient tumor. Both tumors exhibited MSI-L phenotype (Table 3).

**Table 3:** Clinicopathological characteristics and MSI profile of patients with deficient MMR protein

Case N°	Age (years)	Lost MMR protein	Gender	Family/personal history	Grade	TNM Stage	MSI-status	Altered marker
1	56	MSH6	Male	No/no	G3	pT4aN1	MSI-L	D17S250
2	82	MLH1/PMS2	Male	No/no	G3	pT2N0	MSI-H	BAT26

## Discussion

Despite extensive research on MMR and MSI in various malignancies, their roles in bladder cancer remain poorly understood. Given the potential impact of MMR and MSI status on treatment response and patient prognosis, investigating these molecular patterns in bladder cancer is crucial. In this study, we explored the MMR protein expression and the prevalence of MSI in urothelial carcinoma of the bladder, with the objective of providing insights that could inform targeted therapeutic strategies and improve clinical outcomes for affected patients.

Numerous studies have examined MMR protein expression and MSI in bladder cancer. Reported frequencies of MMR protein loss range from 0 to 8%, while MSI prevalence varies between 0 and 100% [23–30]. In our study, only two patients exhibited altered MMR protein profiles, with 95.9% (47 patients) showing intact profiles. Among the altered cases, one patient exhibited a concurrent loss of MLH1 and PMS2, along with instability at BAT26, resulting in an MSI-L profile. The other patient showed an isolated loss of MSH6 and D17S250 instability, leading to an MSI-L phenotype.

Consistent with our findings, earlier studies have emphasized the rarity of MSI in bladder cancer. Gonzalez-Zulueta et al. reported somatic instability in only 3% of 200 bladder tumor samples [29], while Bonnal et al. found no alterations in most of the 33 transitional cell carcinoma (TCC) samples, with only one case showing a variation in the D17S250 marker [30]. Kassem et al. identified a 4% loss of *hMLH1* or *hMSH2* gene expression in TCC cases and an 8% loss of MSH2 in squamous cell carcinoma samples from Egyptian bladder cancer patients [23]. Other studies have also corroborated these findings, reporting low frequencies of MSI and MMR protein loss in bladder cancer. Yamamoto et al. identified MSI-positive status in 9 out of 100 bladder tumors, with reduced MMR protein expression in 6% of cases for MLH1, 5% for MSH2, and 2% for both [25]. Similarly, Giedl et al. documented abnormal MMR expression in 2.1% of younger bladder cancer patients and 6.5% in an unselected cohort [27].

More recently, Hodgson et al. reported that 2% of patients with muscle-invasive high-grade bladder cancer exhibited deficient MMR tumors [31]. Likewise, Fraune et al. [28] and Kagawa et al. [32] observed MMR deficiency in 1.5% of bladder cancer patients from large, unselected cohorts. Fraune et al. identified MSI positivity in five out of nine suspected cases, resulting in a prevalence of 1.1% [28]. Mohamedali et al. found MMR deficiency in 2% of cases from an Eastern Indian population, including one case with loss of both MSH2 and MSH6 expression and another with deficiencies in all four MMR proteins [33].

Furthermore, comparative studies have highlighted differences in MMR protein expression between various forms of urothelial carcinoma. Ju et al. found that MMR protein loss and MSI were more prevalent in upper tract urothelial carcinoma (UTUC) than in bladder urothelial carcinoma, with 9% of UTUC cases showing MMR protein loss compared to only 1% of bladder urothelial carcinomas [34]. Similarly, Necchi et al. reported significantly higher rates of MSI-H status in UTUC (3.4%) compared to bladder urothelial carcinoma (0.8%) [35]. Yang et al. also reported MMR deficiency in 8.89% of UTUC patients, compared to 2.74% in bladder urothelial carcinoma, revealing notable differences in the genomic landscapes and molecular characteristics between these two urothelial carcinoma subtypes [36].

A comprehensive meta-analysis by Chandran et al. further validated these findings, reporting an overall prevalence of dMMR in urothelial carcinoma patients of 6%, with bladder cancer at 2% and UTUC at 9% [37]. The most common patterns of MMR protein loss in bladder cancer were PMS2 (0.5%), MLH1/PMS2 (0.5%), and MSH6 (0.4%) [37]. The prevalence of MSI-H was 3% in urothelial carcinoma, 1% in bladder cancer, and 11% in UTUC, highlighting significant molecular differences between bladder cancer and UTUC. MSI-H was more frequently observed in localized diseases compared to metastatic cases. The study also suggested that dMMR or MSI-H status might be linked to higher sensitivity to immune checkpoint inhibitors and chemotherapy resistance [37].

In contrast, other research has reported higher frequencies of MSI and related alterations in bladder carcinoma. Uchida et al. [38] and Li et al. [39] observed increased rates of dinucleotide repeat alterations, with mutations found in 21% (8/38) and 17% (12/72) of patients, respectively. However, alterations involving multiple microsatellite markers were infrequent, occurring in only 8% and 3% of cases. Additionally, three subsequent studies identified very high levels of MSI. Christensen et al. reported an unusually high 100% instability in 14 bladder cancer cases across various tumor stages and grades [40]. Vaish et al. found a high rate of MSI in 44 TCC patients, with 72.7% of tumors showing MSI [41]. High-stage and high-grade tumors exhibited MSI in 40.6% and 59.4% of cases, respectively, and MSI was associated with tumor recurrence, suggesting its potential as a prognostic marker for superficial bladder tumors. Migaldi et al. documented length variations in 94% of 51 superficial papillary bladder urothelial carcinomas in young patients, linking these alterations to a reduced risk of tumor recurrence and disease progression, highlighting the prognostic value of microsatellite alterations in early-stage bladder cancers [42].

Further studies investigating MSI and MMR protein expression have yielded diverse results. Catto et al. examined 111 TCC patients, analyzing hMLH1 and hMSH2 expression and MSI at 14 loci in 84 tumors [43]. They found reduced MMR expression in 23% of tumors, particularly in muscle-invasive cases, and detected MSI in 8% of tumors, with multiple loci affected in 1% and a single locus in 7% of cases. These findings suggest that decreased MMR expression may contribute to TCC development and could potentially serve as a prognostic marker. Similarly, Seatta et al. reported MSI in 16.6% of 72 primary TCC cases, significantly linked to reduced hMLH1 expression, although MSI did not correlate with tumor grade, stage, or survival outcomes [24]. Reduced hMLH1 expression was identified as a predictor of shorter disease-free survival.

Recent studies have also investigated MSI and MMR in the context of Lynch syndrome. Joost et al. investigated the association between urinary tract malignancies, MSI, and Lynch syndrome, reporting a cumulative risk of 3.3% for bladder urothelial carcinoma in men and 2.6% in women [44]. While only 20% of bladder urothelial tumors exhibited an MSI-H phenotype, 86% showed MMR loss, suggesting that bladder urothelial carcinoma may be classified within the Lynch syndrome tumor spectrum. Similarly, Rasmussen et al. analyzed 97 urothelial tumors from Lynch syndrome carriers and found that 88.7% displayed MMR loss, with 70.6% classified as MSI-H [45]. Sequencing-based analyses using marker panels showed high concordance, although some tumors retained MMR expression despite being classified as MSI-H or MSI-L. These findings highlight the variability in MSI detection methods, which may influence result interpretation and clinical management, underscoring the need for standardized MSI assessment protocols to ensure consistent and accurate diagnostic outcomes [45].

The variation in the frequency of MMR expression loss and MSI in bladder tumors across different studies can largely be attributed to several factors. These include differences in specimen types (such as archived tissue, fresh tissue, biopsies, or tissue microarrays), the methodologies employed (immunohistochemistry, PCR, NGS sequencing), and the specific biomarker panels used. Additionally, discrepancies may arise from variations in the number and sensitivity of microsatellite loci analyzed, tumor heterogeneity (tumor grade and stage), differences in data interpretation, and potential ethnic variations. Certain studies, including those by Kassem et al. [23], Seatta et al. [24], and Burger et al. [26], utilized limited antibody panels (e.g., MLH1 and MSH2 immunohistochemistry) to assess MMR protein loss, which may have resulted in an underdiagnosis of MSI cases. For instance, Catto et al. reported an MSI-H case without corresponding MMR deficiency [43]. Conversely, weak MMR protein staining, misinterpretation of results, and missed positive-staining regions—attributable to tissue heterogeneity, inadequate fixation, or poor sample quality—can lead to inflated reports of MMR loss without corresponding MSI. Fraune et al. noted discrepancies between MMR status in tumor micro-array (TMA) spots and larger tissue sections, where the TMA cylinder often contained regions with reduced immunoreactivity, including areas with weak immunostaining in stromal cells but absent staining in tumor cells [28].

The National Cancer Institute/International Collaborative Group on HNPCC recommended a standard diagnostic procedure that involved analyzing both tumor and normal tissues using five microsatellite markers. These markers consist of two mononucleotide repeats (BAT26 and BAT25) and three dinucleotide repeats (D2S123, D5S346, and D17S250) [16]. Originally developed for detecting MSI in Lynch syndrome-related cancers, this panel has been utilized in several studies, including ours [24, 26, 27]. However, various studies have employed alternative or additional panels, each with differing sensitivities for PCR- or sequencing-based MSI analysis [23, 29, 30, 33–38, 42–45]. This variability in marker selection and sensitivity highlights the need for the development of a bladder cancer-specific panel to enhance diagnostic consistency.

## Conclusion

Although this study is limited by its retrospective design and small sample size due to its single-center nature, it represents the first investigation of MSI and MMR protein status in Tunisian patients with bladder urothelial carcinoma. The detection of MSI-L/MMR-deficient status in only 4.1% of cases underscores the rarity of MSI in bladder urothelial cancer. To expand upon these findings, future multi-center, prospective studies are required to clarify the role of MSI in bladder cancer and to establish a standardized diagnostic approach. Such research could also facilitate the identification of MSI as a potential predictive biomarker for targeted therapeutic interventions in bladder cancer.

## Data Availability

No datasets were generated or analysed during the current study.

## Abbreviations

dMMR: Deficient mismatch repair
FFPE: Formalin-fixed, paraffin-embedded
MLH1: MutL homolog 1
MMR: Mismatch repair
MSH2: MutS homolog 2
MSH6: MutS homolog 6
MSI: Microsatellite instability
MSI-H: MSI-high
MSI-L: MSI-low
MSS: Microsatellite stable
PCR: Polymerase chain reaction
pMMR: Proficient mismatch repair
PMS2: PMS1 homolog 2
TCC: Transitional cell carcinoma
TMA: Tumor micro-array
TNM: Tumor nodal metastasis
UTUC: Upper tract urothelial carcinoma
WHO: World Health Organization
